# Modeling and Simulation of Industrial and Environmental Processes with Membranes

**DOI:** 10.3390/membranes16070225

**Published:** 2026-06-30

**Authors:** José M. Gozálvez-Zafrilla

**Affiliations:** Institute for Industrial Radiophysical and Environmental Safety (ISIRYM), Universitat Politècnica de València, 46022 Valencia, Spain; jmgz@iqn.upv.es

## 1. Introduction

Membrane technologies are a key technique in modern separation because of their flexibility and the intrinsic modularity of membrane processes, which can reduce energy consumption compared to conventional thermal separations and enable separations that are difficult to achieve with other processes [1,2,3]. That is why membrane processes are widely used in many industrial and environmental applications, such as desalination, water and wastewater treatment, gas separation, purification and product recovery, food and beverage processing, pharmaceutical and biotechnological operations, biomedical devices, membrane reactors, environmental remediation, energy-related technologies, and sensors. In all these fields, the use of membranes continues to evolve and expand.

In the membrane field, as in many other scientific and engineering disciplines, modeling and simulation can play a fundamental role. From a scientific perspective, it can contribute to understanding the underlying membrane phenomena. In membrane engineering, when combined with the modeling of other units, simulation supports process design and development, allowing performance optimization and enabling efficient process control. While many other separation processes are often described using equilibrium-based models, membrane systems are inherently rate-based. Membrane modeling requires accounting for complex interactions among membrane material properties, transport mechanisms, hydrodynamics, and operating conditions. In addition, physicochemical processes such as membrane fouling and scaling must be considered for long-term applications. However, the modularity of membrane systems facilitates scaling and integration into larger processes, thereby enabling more manageable simulation.

Process simulators, except in some specific cases, still include limited or simplified membrane unit models. The main reason lies in the complex interactions described above, which make membrane separations highly case-specific. Membrane researchers, therefore, require a thorough understanding of the underlying phenomena in order to develop reliable membrane models. In addition, they must possess solid mathematical skills and design appropriate experiments to obtain model parameters from experimental data.

Consequently, several modeling levels are typically considered. At the first level, modeling focuses on membrane materials and transport at the microscopic scale. Transport and mechanistic models (e.g., solution–diffusion, Donnan–Steric Pore Model (DSPM), and dusty–gas) as well as phenomenological and empirical models (e.g., resistance-in-series, scaling, and deposition models) are commonly used [4]. These models are usually developed through experiments with small membrane samples or through microscale simulations such as molecular dynamics or pore-network modeling.

A second level of modeling accounts for hydrodynamic effects at the membrane module scale, including phenomena such as concentration polarization and axial dispersion. These effects are typically investigated at pilot-plant scale and are increasingly supported by computational fluid dynamics (CFD) [5,6].

At a larger scale, system-level modeling integrates complete processes and plants. At this level, simulation is commonly used for process design and optimization, as well as for integrating membrane units into hybrid systems with other unit operations, such as adsorption, reaction, or distillation [7,8,9].

Programming and well-established numerical algorithms continue to play an important role in solving and exploiting model results. However, new Artificial Intelligence techniques are emerging as a complementary approach. These techniques include artificial neural networks, machine learning methods, genetic algorithms, and data-driven surrogate models [10,11].

Although the benefits of modeling and simulation in membrane science are considerable, simulation results must always be critically assessed against experimental observations, and the effects of uncertainty should be carefully evaluated [12,13,14].

## 2. Overview of Published Articles

This Special Issue includes contributions that address several aspects of modeling and simulation in membrane science and engineering, as discussed in the Introduction. The contributions have been arranged following a progressive thematic logic from fundamental materials, modeling methods, process applications, to integrated systems:

I. Membrane Materials and Transport Mechanisms (contributions (1)–(3));
II. Physicochemical Phenomena Affecting Membrane Performance (contribution (4));
III. Data-Driven Modeling and Artificial Intelligence (contributions (5), (6));
IV. Filtration Process Modeling and Optimization (contributions (7), (8));
V. Desalination and Industrial Membrane Systems (contributions (9)–(11));
VI. Biological Membrane Systems (contribution (12));
VII. Energy Perspective (contribution (13)).

We start with three fundamental studies on membrane structure and transport, which provide a basis for membrane modeling in their respective fields. Contribution (1) investigates the relationship between polymer structure and gas separation performance in poly(arylene ether ketone)s and poly(diphenylene phthalide) membranes. The study provides valuable insights into how polymer molecular architecture influences permeability and selectivity. The work (2) studies hydrogen permeation through palladium membranes. Permeation is analyzed through a combined experimental and theoretical evaluation of Sievert’s law assumptions, providing an improved understanding of hydrogen transport modeling and explaining differences in experimental observations of the Sievert’s exponent. Contribution (3) investigates phenomena in porous media, analyzing the influence of grain membrane geometry on permeability properties in porous stone. It can provide a reference for different grain structures.

Physicochemical phenomena influencing membrane operation are addressed in contribution (4), through the case of reverse osmosis scaling. In this work, calcium carbonate crystallization was investigated using fluorescent-tagged antiscalants. This idea offers a unique opportunity to track the location of scale-inhibitor molecules during calcite scale formation.

The increasing availability of computational tools has led to the development of new modeling approaches based on Artificial Intelligence and on data-driven methods. The potential of applying Artificial Intelligence methods is exemplified in contribution (5). In this work, different control strategies are compared for a peristaltically pumped low-pressure membrane process, demonstrating the potential of advanced control algorithms to improve operational stability in membrane systems. The ideas generated can be used in a batch process to adjust pressure and crossflow velocity to minimize energy required for the concentration stage or to jointly optimize the operation and cleaning phases. In contribution (6), machine learning techniques are used to analyze the factors influencing the gas-separation performance of carbon molecular sieve membranes. The work results highlight the importance of structural characteristics in gas permeability.

The following contributions shift to unit-operation-scale studies focusing on filtration processes and performance optimization. Gas sparging-assisted microfiltration of bacterial cultivation broths is modeled and optimized using response surface methodology and genetic algorithms in (7), while a related study (8) investigated the use of Kenics static mixers combined with gas sparging to enhance crossflow microfiltration performance and mitigate membrane fouling.

Next, contributions expand to system-level simulations of integrated membrane processes for desalination and environmental applications. A hybrid multi-stage flash–reverse osmosis desalination system is modeled and structurally optimized in (9) to improve system performance. The work provides detailed model-based numerical calculations useful for design. In the work (10), modeling and operational optimization were applied to reverse osmosis for treating wastewater from coal-fired power plants. The work is a significant effort in the development of zero-emission wastewater treatment systems. Another system-level study (11) examines atmospheric water generation via reverse osmosis technology under different flow configurations. The work determined the reverse osmosis membrane configuration that required lower energy demand and stages.

Contribution (12) is the representative of hybrid biological membrane systems in this Special Issue. Microbial metabolic processes in a hydrogen-based membrane biofilm reactor are modeled to evaluate simultaneous bromate and nitrate reduction, highlighting the importance of coupling biological and transport phenomena in environmental treatment systems.

Finally, a comprehensive review of renewable hydrogen production technologies is presented in (13). The review discusses the main production methods, scale-up challenges, and the role of hydrogen in future energy systems, providing a broader context for membrane-based technologies increasingly employed in hydrogen purification and separation processes. This review has received more than 325 citations at the time of the publication of this Special Issue.

## 3. Conclusions

Overall, the contributions to this Special Issue are representative examples of the main modeling approaches used in membrane science and engineering. From fundamental material studies to process simulation and optimization, these works demonstrate how these techniques can contribute to the development and performance optimization of membrane technologies. To date, the average number of citations of the contributions to this Special Issue is higher than the journal average, indicating strong interest in membrane modeling and simulation and highlighting the quality and impact of the published works.

These developments suggest that modeling, simulation, and data-driven methods will continue to play a central role in the future advancement and industrial implementation of membrane technologies.
